# The state of play for contact training and coaching in women's rugby

**DOI:** 10.1002/ejsc.12119

**Published:** 2024-05-10

**Authors:** Anna Stodter, Kathryn Dane

**Affiliations:** ^1^ Carnegie School of Sport Leeds Beckett University Leeds UK; ^2^ Discipline of Physiotherapy Trinity College Dublin School of Medicine Dublin Ireland

**Keywords:** coaching, gender, injury & prevention, training

## Abstract

This article aims to review and comment upon the current “state of play” for research around contact and tackle training in women's rugby, covering tackle injury risk, match contact demands, players' experiences of contact coaching and contact skill preparation. In women's rugby, the tackle is the most common match technical‐physical contest, accounting for around two‐thirds of all injuries and carrying the greatest injury burden. Players' experience and technical abilities are key determinants of tackle safety and performance. Despite this, there is limited research available that connects insight into women's rugby contact demands with the *how* and *why* of effective tackle and contact training and coaching in context. This review suggests that adapting and adopting progressive tackle skill training frameworks and gender‐responsive coaching practices can aid tackle skill learning in women's rugby. Creative transdisciplinary research and more effective translation and implementation activities that take place within the vibrant and growing context of women's rugby can enhance science and safety whilst working as a medium for social change.

## Highlights

- The ability to tackle and be tackled is perhaps the most essential contact skill required for successful rugby participation. The practice and professional development of coaches to encourage safe and effective tackle technical actions is an important priority in women's rugby.
- Existing work has taken a sex‐specific approach, largely failing to engage with coaches, athletes, their experiences and wider context, which limits successful implementation and progress.
- We suggest that a more collective, gender‐responsive and evidence‐informed approach that includes coaches and players is needed to effectively “tackle” women's rugby safety, performance and coaching development. Our recommendations are mapped to the socioecological structures (individual, interpersonal, organizational and societal) within which players and coaches operate.

## 1 INTRODUCTION

Women's participation in the contact sports of rugby union and rugby league is increasing globally alongside a rapidly changing professional landscape (The Rugby Football League, 2021; World Rugby, 2019a). Spurred on by the inclusion of rugby sevens in the 2016 Rio Olympics, there is growing interest and investment in women's participation across the codes of union and league (Scantlebury & Heyward, 2022). With a global participation of 2.7 million players, women's rugby union is growing by approximately 28% per year alongside current targets to close the gender gap in better supporting women and girls (World Rugby, 2023c). Rugby league shows relatively smaller global participation numbers, yet the introduction of top tier competitions such as the English Women's Super League in 2017 and the Australian National Rugby League Women's competition in 2018 means that continued growth is occurring through and beyond playing. Accordingly, 99% of players, coaches and volunteers want to see more effort go into growing and supporting women's rugby league (The Rugby Football League, 2021). Across the codes, rugby is a highly complex, fast paced contact game requiring a broad skill set that includes tackling, rucking, passing and catching. Success is dependent partly on a team's defensive strategy and contact proficiency (Scott et al., 2023). In addition, the tackle event is consistently reported to be responsible for the highest number of injuries in union and league (McLeod et al., 2023; Starling et al., 2023). A major risk factor for tackle injury and a key determinant of tackle performance is the player's technical ability (Burger et al., 2020; McLeod et al., 2023). In other words, the ability to tackle is one of the most essential contact skills required for safe and successful rugby participation. Ball carrying in contact, or being tackled, may also be particularly important in women's rugby where research has tentatively suggested that head to ground contact on landing from the tackle possesses a high propensity for head impact events (McLeod et al., 2023) linked to uncontrolled whiplash movements (Williams et al., 2022). Technique is influenced by playing experience, and it is possible that women rugby players may have limited opportunity to participate in the physical contact and collisions inherent in tackle situations (Dane et al., 2022; Peck et al., 2013). This results in a relatively low “training age” in women rugby players, who may also have minimal access to facilities and quality support (Brown et al., 2023; Heyward et al., 2020). Alongside this, performance in the tackle and positive attitudes toward injury prevention are associated with coaches' behaviors and use of learning resources to promote proper technique (Hendricks et al., 2020). As such, the practice and professional development of coaches to encourage safe and effective tackle technical actions remains an important priority through law changes (World Rugby, 2023a), contact load guidelines (World Rugby, 2022), and the promotion of educational tackle safety programmes (e.g., Tackle Ready & Contact Confident; World Rugby, 2023a, 2022). Despite the assumed benefits of these initiatives, influential aspects such as coaching perspectives and considerations can often be an afterthought, as well as the wider gendered social and cultural environment within which women's rugby takes place. While systematic reviews benefit from drawing upon numerous quality published research articles to draw robust conclusions, very few studies directly investigate preparatory strategies and coaching in women's rugby (Dane et al., 2022; Paul, Davidow, et al., 2023) or successfully translate findings to practice. The aim of this article therefore is to critically review existing literature around tackle training in contact rugby codes and distil some key messages for coaches and related stakeholders to use as part of a transdisciplinary approach in enhancing the science and safety, and importantly, the gender equity of women's rugby.

## 2 THE STATE OF PLAY: A RESEARCH OVERVIEW

The limited research on tackle and contact training in the context of women's rugby has focused largely on injury risk and prevention (Burger et al., 2020). While wider women's sport research has emphasized intrinsic sex‐based biological views addressing anatomy, physiology and hormones, recent perspectives have begun to embed powerful gendered social influences in the study of contact sports (Fox et al., 2020). For example, investigating anterior cruciate ligament injuries and concussion in women's Australian Rules Football (AFL; e.g. Bruder et al., 2023; Fox et al., 2020). Accordingly, in this review we saw it as important to recognize the differentiation between sex (a complex blend of physical and physiological characteristics) and gender (identities, roles and structures formed by society) as separate and non‐binary concepts (Parsons, Cohen, & Bekker, 2021). When discussing sex‐specific factors then, the term “female” is used, otherwise we adopt the all‐encompassing term 'women', referring to adults, throughout. Placing women's bodies, their training and coaching in context by acknowledging the gendered factors and environments that characterize the state of play, we are better positioned to understand and achieve more equitable outcomes in women's rugby (Parsons et al., 2021).

### 2.1 Tackle injury risk

In women's rugby union, the tackle is the most common match injury event (11.2 per 1000 h; West et al., 2021a) and carries the greatest burden of injury (615 days absence per 1000 h; Starling et al., 2023). Although tackles in women's rugby account for a greater proportion of injuries than in men's rugby (67% vs. 43%), the incidence (21.6 per 1000 h) and burden of tackle‐related injury (987 days absence per 1000 h) are lower than that of elite men's rugby (West et al., 2021b). Similarly, in rugby sevens and rugby league, the proportion of tackle‐related injury is 62% (Fuller & Taylor, 2021) and 80% (King & Gabbett, 2007), respectively. In women's sports, significant emphasis has been placed on the role of female biology as a primary determinant of injury risk (Fox et al., 2020). It is well established that female athletes have hormonally mediated distinctions from male athletes, including characteristics like the menstrual cycle, breasts and pregnancy. These sex differences can impact contact injury risk and performance, but it is important to acknowledge that hormonal fluctuations and physiological distinctions are not the only contributors (Parsons et al., 2021). Restricting women's tackle injury risk to biological causes (e.g., biomechanics, hormones, physiology), which are generally unchangeable, may perpetuate constraining and harmful gender stereotypes, may be misrepresentative of the biggest issues and likely overlooks the complex sociological structures that shape the performance environment of rugby (Cummins et al., 2020; Fox et al., 2020; Parsons, Cohen, & Bekker, 2021). This is part of a wider bioscientific, medicalized discourse (e.g., Heyward et al., 2022) which positions women as a homogenous body of docile subjects of study, not “naturally” fit for contact sports such as rugby (Parsons, et al., 2021) or enriched by diverse intersectional experiences.

A gendered environmental approach acknowledges the sociocultural and environmental conditions that contribute to injury risk and better accounts for women's experiences in this space (Fox et al., 2020; Parsons, et al., 2021). More so than most sports, rugby is a deeply gendered environment, where participation, coaching, support, administration, resources and opportunities have long been dominated by men (Joncheray, 2021). While sex differences are not modifiable, influential aspects of the environment within which women play rugby, such as societal expectations of how women should use or move their bodies, and access to appropriate facilities, training and medical support, can be changed for the better. For example, the risk of injury from tackling can be mitigated through the implementation of a safe and effective technique that is largely developed during training and supported through coaching input (Hendricks & Lambert, 2010). Coaches and coaching are a key aspect of the women's rugby environment that is often neglected in research. There is also a disconnect between the technocratic rationality furthered by sterile, controlled, bio‐scientific knowledge and its implementation through coaching practice which is highly relational, dynamic and context‐specific (e.g., Bowes & Jones, 2006; Lyle, 2018). Taking a different approach that connects layers of sport science, evidence and research with coaching considerations and literature can afford previously unseen benefits for the unique context of women's rugby.

### 2.2 Gender research gap

While the participation and professionalism of women rugby players has witnessed significant growth, the associated research literature and applied practice still lag behind that of men's rugby. There is a lack of directly relevant research evidence available to inform contact training and coaching in women's rugby (Hendricks et al., 2023), for example, in relation to physical (Heyward et al., 2020; Scantlebury & Heyward, 2022) and technical preparation (Dane et al., 2022). Overall, less than 2% of coaching research in rugby is conducted with women (Paul, Davidow, et al., 2023). This has led to male‐derived training and coaching practices based on assumptions, tradition and myths that may be problematic in their application to women's rugby players and contexts (Dane, Foley, Hendricks, & Wilson, 2023; Ross, 2023). The situation is reflective of women's sport in general, where a lack of research attention (Cowley et al., 2021) and ingrained stereotypes and gendered norms mean athletes can mistakenly be thought of as “a different kettle of fish” (Jones & Avner, 2021). In other words, the inherently “different” or “puzzling” nature of female athletes, positioned in comparison to the default male, is thought to present an “extra challenge” for effective coaching. Such assumptions can unintentionally embed rather than challenge sex inequalities in sport science and coaching (Malcolm, 2023), reinforcing the need to problematize accepted “truths” and draw upon wide ranging types of evidence to inform gender‐responsive coaching practice (Jones & Avner, 2021).

### 2.3 Match contact demands

One readily available form of evidence that can inform contact training and coaching in women's rugby relates to the demands of the game. Contact events in rugby match‐play require the integration of strength, endurance, agility and speed to get into optimal positions for executing forceful full‐body collisions. These contact events aim to impede opponents' progress in defense or maintain and advance possession toward the try‐line in attack. Successful contact performance in rugby requires that players are attuned to relevant information unfolding during contact events (e.g., *ball‐carrier or tackler behavior, field position, approach height and speed*) and can detect and use this information to guide technical/tactical actions to achieve performance goals (Araújo et al., 2005; Passos et al., 2008). Simultaneously, players must also navigate the perpetual risk and potential fear of injury stemming from the dynamic impact of collisions (Dane et al., 2023b). Traditionally, there have been assumptions about the existence of “textbook” tackle techniques and “optimal movement patterns” for safe and effective contact. However, scholars have cautioned against “one size fits all” coaching methods for technical/tactical skills such as tackling (e.g., Passos et al., 2008). This is because live match‐play in competitive team sports is not a stable context in which information is certain. There are also differences in body mass and muscular distribution between male and female players, so “optimal” tackle technique may vary (Daly et al., 2022). According to Newell (1986), players' technical/tactical actions are defined by the interactions of (i) their own individual characteristics (e.g., psychological states, physical, technical and tactical attributes, etc.); (ii) the task characteristics (e.g., teammates involved, opposition behavior, etc.); and (iii) the environment characteristics (e.g., weather, playing surface, spectators, etc.). Therefore, it is important for rugby coaches to understand the contact demands and goals of the game to enable them to systematically plan and execute training activities that holistically emphasize the physical, technical, tactical and psychological abilities and principles the players need to employ (Hendricks et al., 2018, 2023; Tee, Ashford & Piggott, 2018). Rugby requires players to engage in periods of high‐intensity activity (e.g., sprinting, tackling, rucking and scrummaging) separated by periods of lower intensity activity (e.g., standing, walking and jogging; Dane et al., 2022). Women's rugby players need a capacity for intermittent endurance, repeated sprint ability, accelerations and decelerations and collision‐based exertions. The tackle contest is the most common match technical‐physical contest with up to 280 tackles on average per game (West et al., 2022). Contact demands vary by level of competition, rugby code (e.g., league, union, sevens) and playing position. Players need to be physically, technically, tactically and psychologically prepared to execute contact demands under fatigue and pressure, and coaches play a key role in this preparation (Tee et al., 2018).

The rapid growth and professionalism of women's rugby mean that coaches are invested in developing and applying knowledge to advance the contact aspects of the game (e.g., defensive strategy and tackle technique) to gain competitive advantages over opposition. Consequently, the game has become more physically and technically demanding, leading to a heightened focus on skill development supported by performance analysis, sports science and strength and conditioning. Research on the technical‐tactical demands of rugby typically provides information on the frequency of technical actions and events. Knowledge of the average demands of match‐play can be used by coaches to enhance and support player development by planning and delivering appropriately aligned contact training practices. Drawing on match data from three playing seasons (2017, 2018 and 2019), Woodhouse et al. (2021) revealed that international women's rugby union players can be exposed to between 0.91 and 1.43 collisions per minute, depending on the playing position. Across 80 min of match‐play, collisions per player are higher among forwards (range: 18.3–37.2 collisions) than backs (range: 7.5–14.3 collisions; Woodhouse et al., 2021), indicating a need for position‐specific consideration, planning and delivery of technical‐tactical contact training.

Whether as a ball‐carrier or tackler, rugby tackle contact success involves a set of highly technical and physical movement skills. Tackle technique refers to the execution of a set of coordinated movement patterns, while the skill of tackling refers to the proficiency of execution of the correct actions in response to the demand of the situation (Hendricks et al., 2018). Like any skill, coaching correct contact technique and capacity through training is crucial in enhancing performance and reducing injury. Within given time constraints, there is a need for efficient contact training practices that provide concurrent development of the physical, technical and tactical elements of tackle contests. World Rugby's contact load guidelines recommend an upper limit of 40 min of controlled contact training and 15 min of full contact training per week for professional athletes (World Rugby 2023b). Regular weekly exposure to both types of contact training offers opportunities for the acquisition or refinement of contact skills. Coaches are responsible for promoting players' understanding of where and why contact skills should be employed not just the what and the how (e.g., contact technique). As such, contact training environments should aim to promote the frequency and quality of contact involvements under conditions that are representative of match demands. Remaining cognisant of World Rugby recommended contact loads, video‐based technical feedback can improve tackling technique and coaches can use video analysis to monitor tackle technique in training and matches (Davidow et al., 2023; Kerr et al., 2018; World Rugby 2023b).

While useful in providing insight into *what* to coach (e.g., match demands and performance analysis knowledge), the developing literature in women's rugby does not consider the *how* or *why* behind effective contact coaching (e.g., integrating coaches' pedagogical, interpersonal and intrapersonal knowledge; Côté & Gilbert, 2009). Research has also failed to apply fragmented bio‐scientific knowledge (such as male‐derived performance analysis data on head contact mechanisms in elite rugby league; McLeod et al., 2023) to the socio‐cultural contexts of women's rugby coaching at different levels. For instance, there is a large gap between understanding head contact mechanisms in elite men's rugby league to supporting coaches' ability to deliver safe and effective contact coaching to groups of women rugby players. Add in these players' likely mixed training and gym experiences, limited contact sport backgrounds, and varied ages and abilities, and some of the coaching challenges which greatly influence contact skill preparation begin to become apparent (Dane, Foley, Hendricks, & Wilson, 2023, 2024; Nolan et al., 2023; Peck et al., 2013). While women's and men's rugby contexts may share performance principles, these principles need to be tailored to suit the training environment, performance level and needs of the players. The current literature largely fails to recognize coaching as a complex pedagogical and social practice that is relationally interdependent and shaped by particular cultural and historical contexts. Some relevant factors to consider might be communication patterns in pre‐season training versus competitive match situations (Mouchet, Harvey & Light, 2014); women's professional sports continuing to be considered second class to men's sports (Lebel et al., 2021); or the longstanding oppression and marginalization of intersectional identities in and through sports (Casey et al., 2022). In not acknowledging these and other important factors therefore, research has remained largely misaligned to the effective coaching of women rugby players.

While few would argue against the need for evidence‐informed approaches that can apply, implement and maintain useful contributions from sport science and medicine, coaching and coaches are often neglected or not involved in the research process. Little is known about the knowledge, experience, qualifications and practices of the women's rugby coaching workforce (Brown et al., 2023). Indeed, coaching behavior is one influential and controllable aspect of the gendered environment within which women play rugby, so it is important to get coaches' involvement and development right. Although women's rugby coaches exhibit strong intentions (e.g., high awareness of tackle injury risks and goals to coach safe contact techniques), there is a gap between intentions and ability to execute these goals in practice (Dane et al., 2024). Often, coaches—who are time‐poor and overwhelmingly volunteers—are tasked with delivering complex injury prevention initiatives developed and tested in male contexts (e.g., Activate; Barden et al., 2022) with minimal preparation or support around how. Meanwhile, “increased coach education” is recommended as a seemingly straightforward solution to the contact‐related ailments of women's rugby including heightened injury rates, greater injury burden and poorer injury outcomes than in men's rugby (e.g., Scantlebury & Heyward, 2022), with little consideration for coach learning principles or impact on coaches and athletes. Generally, this situation in women's rugby is reflective of a remaining gap in translating sport science research into practice with key stakeholders such as coaches and athletes (Fullagar et al., 2019). A more collective evidence‐informed approach that includes coaches and players is needed to effectively “tackle” women's rugby safety, training and coaching development (Hendricks et al., 2023).

### 2.4 Coaching and coach development in women's rugby

It is generally assumed that coaches' knowledge and implementation of effective contact coaching processes aligned with the context of women's rugby can be cultivated through coach education and development. While coach learning is influenced by a complex, idiosyncratic blend of experiences in formal, non‐formal, and informal development situations (Nelson, Cushion & Potrac, 2006), the impact on coaches' knowledge and behavior (implementation) remains equivocal (Stodter & Cushion, 2019). Formal learning situations are structured, mediated, and often mandated (e.g., coach education courses leading to qualifications), while non‐formal learning situations are structured, mediated, but coach‐initiated (e.g., regional contact coaching clinics), and informal learning situations are unstructured, unmediated, and coach initiated (e.g., learning from observing other coaches). Research in men's rugby shows that coaches prefer to draw on their own playing experiences and informal learning from others as valued sources of contact coaching knowledge (Hendricks & Sarembock, 2013). However, given the likely prevalence of myth, misconception and misogyny surrounding these sources, formal and non‐formal learning situations offer the potential to develop a more quality‐controlled, evidence‐informed, theoretically underpinned and up‐to‐date knowledge base. Research suggests there is a lack of formal education regarding the preparation of athletes in women's rugby (Nolan et al., 2023) alongside a need for improvements to provision to help address the under‐representation of women rugby coaches (Barrett et al., 2021). Women remain peripheral figures on the coaching landscape (Norman et al., 2018), and in rugby, their progression to higher qualification levels is limited (World Rugby, 2020). Women in coaching may not enjoy similar access to informal networks or support structures as men, meaning there is not only an opportunity to provide more help in this area but also to develop innovative coaching knowledge and practices that do not simply replicate and reinforce accepted traditions. To ensure new contact coaching knowledge progresses and is accessible, relevant and applicable to “real‐world” women's rugby contexts, the design of coach learning opportunities is an important consideration that has often been neglected. Impactful opportunities would address *what* and *how* to coach, recognizing coaches' existing knowledge and practice and using cycles of reflective conversations to help coaches make meaningful connections to implementation in context (Paul, Isaacs, et al., 2023; Stodter & Cushion, 2017).

### 2.5 Players' experiences of contact coaching in women's rugby

As part of a collective approach, acknowledging and integrating athletes' experiences can help practitioners to be more “athlete centered” with player welfare at the forefront of contact coaching (Mouchet et al., 2014; Paul, Davidow, et al., 2023). An important contribution to this area has come from scholars drawing on lived experiences to understand the inter‐related factors, actors and cultures that shape tackle injury prevention and skill learning (Dane et al., 2023a, 2023b). Despite the tackle technique coaching resources available (e.g., Tackle Ready; World Rugby, 2019b), how these ideas play out “on the ground” and the extent to which players and coaches engage with recommendations remain underexplored. Adherence is likely to be shaped by the socio‐cultural‐historical constraints within the rugby performance context and the multifaceted interactions between players, coaches, and behavioral opportunities provided in practice. While women rugby players' strong social identities evidently perpetuate certain cultures, for example, around concussion disclosure (Ryan, Daly & Blackett, 2023), coaches serve as powerful influencers on players' beliefs and behaviors and can facilitate positive attitudes, skill learning and performance through their language and actions (Hendricks et al., 2020). Yet women rugby players have reported ineffective or “the bare minimum” standards of tackle coaching (Dane, Foley, Hendricks, & Wilson, 2023). Dane et al. (2023a, 2023b) recently highlighted that maladaptive tackle coaching can have a detrimental effect on women rugby players' skill development and perceived match preparedness, akin to “being thrown in at the deep end.” Tackle training approaches that women rugby players reported as important for their development and safety included individualized progressive structured tackle training plans, using video analysis, adopting adjuncts (e.g., tackle mats), using consistent and memorable instruction, allowing time for questions, and collaborating with external expertize (e.g., mixed martial arts, judo and guest coaches). Players also revealed the emotional investment in tackle training in terms of affording additional time for contact skill practice to build confidence, helping them understand the “why” behind tackle training and promoting player input (Dane et al., 2023a). Additionally, the relational expertize of coaches is often at the forefront of women's coaching needs (Norman, 2016). Although culturally learned dispositions shape tackle beliefs and behaviors, research demonstrates the importance of gender‐responsive coaching language and practices that move away from embedding norms of men's rugby in tackle skill learning in women's rugby (Dane et al., 2023a; Norman, 2016).

### 2.6 Contact skill preparation

**FIGURE 1:**
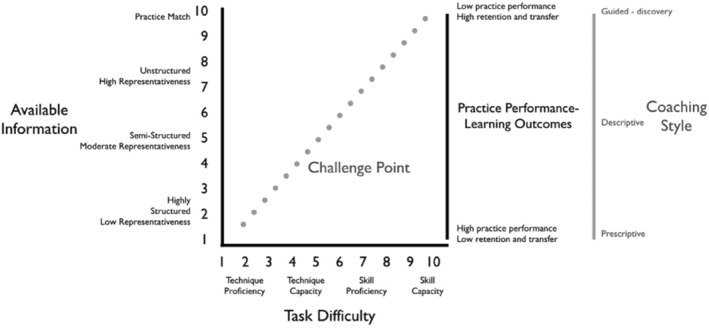
Technical skill training framework (adapted from Hendricks et al., 2018).

Although not specific to women's rugby, Hendricks et al. (2018) proposed a tackle skill training framework that uses skill acquisition literature to allow coaches to plan for tackle training that is modifiable to the team setting, the technical ability and skill level of players, and the time of the season. The framework also outlines skill workload measurements to evaluate the intensity of training (according to task difficulty, rating of perceived challenge and skill load) and suggested aligned styles of coaching. For example, for a progressive contact skill training plan, it is recommended to start with a low challenge point (high levels of technique performance in highly structured practice, utilizing a prescriptive coaching style) and build toward a high challenge point (skill capacity in highly unstructured practice with a guided discovery coaching style) (see Figure 1).

Hendricks et al. (2018) suggest coaches use these variables to divide training purposes into (1) learning tackle contact technique(s) (e.g., (when introducing new players to rugby); (2) developing and refining technical proficiency; (3) building technical capacity (i.e., the maintaining quality technique under a fatigued state); (4) developing and refining tackle contact skill proficiency (quality); and (5) building skill capacity (i.e., the ability to maintain quality skill under fatigue). The framework may also be adapted for contact readiness, for match warm‐ups, and return to contact for players after injury or long periods out of the game (e.g., concussion, postpartum). While this technical skill training framework provides valuable parameters for coaches to adapt their contact training practices to the context of women's rugby, it remains physically and tactically/technically focused without attention to psychosocial factors or connections to wider player welfare. Women play contact rugby because they value the challenge and physical nature of contact, they enjoy the game and they like the social camaraderie (Joncheray, 2021). While a few athletes might transition from community rugby to the elite or rapidly progressing professional levels, the majority will not. Irrespective of the level of the game that a rugby player reaches, it is imperative that they can access supportive coaching environments that promote physically and psychologically safe development, enjoyment and a lifetime of positive engagement in the sport (e.g., England Rugby Development Framework, 2021).

Recent initiatives such as World Rugby's Women's Player Welfare Working Group have attempted to promote evidence‐informed recommendations and interventions specifically for the women's game at all levels, including the development of women's contact coaching. This work supported the generation of “Contact Confident,” a co‐produced resource using evidence and current practice from women's rugby. This freely available online resource comprises a set of videos outlining training activities with Key Performance Indicators for coaches, designed to promote confidence, physical competence and safety in the contact area (World Rugby, 2022). “Contact Confident” forms part of a wider “Tackle Ready” program and is named as such based on case‐study data where collegiate women rugby players reported valuing opportunities to enhance the psychosocial aspects of contact skill development. Coaches too noted the importance of their own responsibilities and confidence delivering contact training (Stodter, 2023; Stodter, Lillis & McDonald, 2023).

## 3 RECOMMENDATIONS FOR RESEARCH AND PRACTICE

**FIGURE 2:**
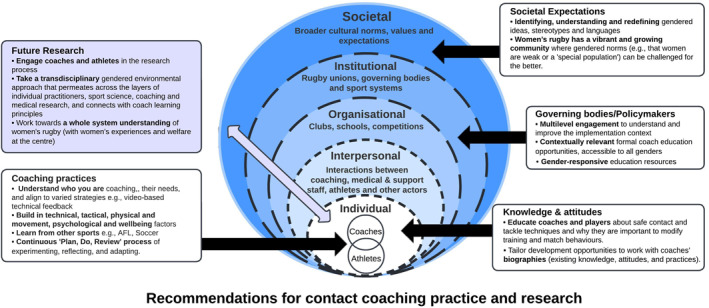
Recommendations to support contact coaching practice and research in women's rugby union applicable to the socio‐ecological model (adapted from Bronfenbrenner, 1979).

Having provided a brief narrative review of the state of play in women's rugby contact coaching literature, we outline some key messages and recommendations aimed to provide safer, more fulfiling and enjoyable contact participation and performance for women rugby players. Our recommendations can be effectively mapped to the socioecological structures (individual, interpersonal, organizational and societal) within which players and coaches operate, influencing contact and coaching behaviors, performance, safety and development (Bronfenbrenner, 1979; Hendricks et al., 2023) (see Figure 2). While tidy two‐dimensional diagrams that simplify complexity are usually deceptive, we believe that mapping out the (permeable) layers of influence acts as a useful reminder to seek and locate understanding across disciplines for the advancement of women's rugby and rugby as a whole.

## 4 ATHLETES AND COACHES

- **Knowledge and attitudes**
  - Educate athletes and coaches about safe contact and tackle techniques and why they are important to modify training and match behaviors.
  - Tailor development opportunities to work with individual coaches' biographies (existing knowledge, attitudes and practices).
- **Coaching practices**
  - **Seek to understand who you are coaching**, their stage of development, individual needs (e.g., differences in playing experiences and abilities) and align to varied strategies, for example, video‐based technical feedback, adapting the tackle skill training framework to plan and progress contact skills (Hendricks et al., 2018).
  - **Build in technical, tactical, physical and movement, psychological and wellbeing** factors across a long‐term curriculum to shape skill development and the athlete experience more holistically.
  - **Learn from other sports** and coaches and adapt to the specific women's rugby context. For example, “Prep to Play PRO” is an athlete preparation resource developed through multiple stakeholder engagements in women's Australian Rules Football (Bruder et al., 2023). Meanwhile, in soccer, UEFA has created a Women's Football Competence Framework for coaches providing some evidence‐based “food for thought” (UEFA, 2023, p. 2).
  - **Employ a continuous “Plan Do Review” process** of experimenting, reflecting and adapting based on the alignment between coaching practice expectations and the realities of athletes' experiences.

### 4.1 Governing bodies/policymakers

- **Multilevel engagement to understand and improve the implementation context.** To modify players' contact behaviors, it is crucial to involve players and coaches to better understand their experiences of women's rugby contexts. Institutions should question how to address inequities for the benefits of athletes (Fox et al., 2020) and consider changing policies and systems to create more equitable and culturally inclusive climates. For example, actively engaging with athletes to design coach development, supporting women through formal coach education and addressing the “leaky pipeline” around coaching opportunities.
- **Contextually relevant formal coach education** opportunities that are accessible to all genders can better contend with the situational realities of coaching.
- **Gender‐responsive education resources.** Develop resources that promote gender‐responsive coaching language and practices that are less concerned with furthering outdated, male‐derived ideas about rugby and more concerned with relevance to the context of women's rugby. Some examples include:
  - Women and girls health and welfare resources by the Rugby Football Union (Rugby Football Union, 2023). While these are not coaching‐specific, educational resources should include all aspects of rugby injuries (Paul, Davidow, et al., 2023). Multi‐component, exercise‐focused interventions have a dose‐response relationship with women's injury rates (Crossley et al., 2020).
  - It is important to consider the characteristics of and wider influences upon women rugby players in order to design, develop and deliver successful contact skill programmes. For example, World Rugby's “Contact Confident” resource outlines evidence‐informed coaching activities specifically co‐produced with athletes and coaches to promote confidence, physical competence and safety in the contact area bespoke to women's rugby (World Rugby, 2022).

### 4.2 Future research

- **Engage coaches and athletes** in the research process. More empirical, coaching practice‐linked research is required to better understand how different approaches impact player outcomes such as contact skill development and injury prevention strategies in women's rugby, acknowledging the important influence of context. As coaching is highly context‐specific, there is no “one right way” to coach, but evidence should inform effective approaches. This can be achieved through embedding researchers in context, overcoming blind spots by inviting and understanding varied stakeholders' perspectives (Lebel et al., 2021) and by reconsidering traditional academic processes to co‐produce knowledge (Smith et al., 2023).
- **Take a transdisciplinary** gendered environmental and socioecological approach that permeates across the layers of individual practitioners, sports science, coaching and medical research and connects with coach learning principles to impact on practice behaviors and positive player outcomes. Many aspects of coaching cannot be assessed using a “traditional” bioscientific research design (Hendricks et al., 2023). Not everything that can be measured counts and not everything that counts can be measured, which presents an inviting challenge for creative transdisciplinary research. Pathways to advance this kind of work include creating opportunities that bring together people from different disciplines, and “zooming out” to challenge, ask others what's missing, and come up with alternative framings (Lebel et al., 2021).

Paradoxically, research exploring sex‐based differences risks reinforcing and extending historical tendencies to “other” female participation in sport (Malcolm, 2023). Research that works *with* and *for* women is still scarce yet vitally important in the context of women's rugby. Rather than falling victim to negativity bias, paying attention to the behaviors and circumstances surrounding “bright spots,” where things are working well, can pique interest, lead us to rethink our approach to women's sport scholarship and impact practical change (Lebel et al., 2021). Sports science and medicine should strive to **understand the whole system of women in rugby** (with women's experiences and welfare at the center).

### 4.3 Societal expectations

- **Identifying, understanding, and redefining** gendered ideas, stereotypes and languages (Norman, 2016).
- Saying that rugby is a risky game for women may be a way to control them socially or at least to soften the disruption of the traditional gender hierarchy (Joncheray & Tlili, 2013). **Women's rugby has a vibrant and growing community** where gendered norms (e.g., that women are weak or a “special population”) can instead be challenged for the better in working toward gender equity.

## 5 CONCLUSION

This review aimed to synthesize and comment on the ‘state of play’ for research around contact training and coaching in women's rugby, leading to some key messages and recommendations mapped onto a bio‐ecological system. While sparse research has primarily addressed female‐specific injury surveillance, prevention, and match demands, recent work has begun to acknowledge the gendered sociocultural and environmental conditions that influence women's experiences of rugby contact and coaching. Research supports the idea that women's rugby contexts are not a microcosm of men's rugby so it is problematic to cut, copy and paste coaching practices based on the accepted tradition or myth (e.g., Dane et al., 2023a). Rather, women's rugby should be understood and coached in a context‐specific manner that integrates the dynamic needs of players, coaches and the training environment. In supporting contact coaching content, that is, “what” to coach, sport science and medicine research need to connect with coach learning principles to meaningfully impact on coaches' knowledge and practice behaviors and lead to positive player outcomes. Researchers in each discipline must strive to work across levels, with coaches and athletes, to understand the whole system of women in rugby (with women's experiences and welfare at the center) and question how to address inequities for the benefit of athletes (Fox et al., 2020).

Rather than presenting a distinctive “challenge” or a negatively framed issue for undertaking and applying research in female sport (Emmonds, Heyward & Jones, 2019), we see this as an opportunity to examine “bright spots” and gain new perspectives (Lebel et al., 2021). Identifying, understanding and redefining gendered ideas, stereotypes and languages and looking outside of our traditional academic frame can help us move beyond the status quo (Norman, 2016). As such, creative transdisciplinary research and more effective translation and implementation activities taking place within the vibrant and growing context of women's rugby can be used as a medium or vehicle for social change. In other words, we hope these messages and recommendations spark new efforts and innovations in the quest to provide more positive, safer and more equitable contact sport participation and performance for women. Ultimately, making things better for women means they will also be better for all.

## CONFLICT OF INTEREST STATEMENT

No conflict of Interest to disclose.
